# Rapid and Safe Isolation of Human Peripheral Blood B and T Lymphocytes through Spiral Microfluidic Channels

**DOI:** 10.1038/s41598-019-44677-3

**Published:** 2019-05-31

**Authors:** Po-Lin Chiu, Chun-Hao Chang, Yu-Ling Lin, Ping-Hsien Tsou, Bor-Ran Li

**Affiliations:** 1 Institute of Biomedical Engineering, College of Electrical and Computer Engineering, National Chiao Tung University, Hsinchu, Taiwan; 2 Agricultural Biotechnology Research Center, Academia Sinica, Taipei, Taiwan; 3 Department of Biological Science and Technology, College of Biological Science and Technology, National Chiao Tung University, Hsinchu, Taiwan; 4 Division of Chest Medicine, Department of Internal Medicine, National Taiwan University Hospital, Hsinchu, Taiwan; 5 Center for Emergent Functional Matter Science, National Chiao Tung University, Hsinchu, Taiwan

**Keywords:** Lab-on-a-chip, Biomedical engineering

## Abstract

Peripheral blood lymphocytes (PBLs) are mature lymphocytes that circulate in the blood rather than being localized to organs. A reliable label-free collection approach that can viably and appropriately isolate PBLs to establish *in vitro* culture systems is crucial for basic research and clinical requirements. However, isolation of PBLs from whole blood is difficult, and so the development of a rapid and safe method to perform this task is required. Microfluidic technology offers opportunities that challenge the performance of macroscale methods. In this study, we proposed a simple spiral microfluidic chip for efficient and high-throughput isolation of lymphocytes from a sample with prelysed RBCs. This spiral microfluidic platform does not rely on antibodies or biological markers for labeling cells of interest while isolating lymphocytes but rather enriches B and T lymphocytes through the different physical properties that are intrinsic to lymphocytes and other blood cells. The device was used to achieve high-throughput (~1.3 × 10^5^ cells/min) separation of lymphocytes with high viability (>95%). Compared with previous approaches, our device provided rapid, label-free, high-throughput, and safe lymphocyte separation.

## Introduction

Peripheral blood lymphocytes (PBLs) are mature lymphocytes that circulate in the blood rather than being localized to organs. They are one of several types of white blood cells (WBCs) that are crucial for the immune system. PBLs comprise B cells, T cells, and natural killer cells^1,2^. B lymphocytes recognize antigens and produce antibodies to fight them^3^. T cells have distinct crucial functions in cell-mediated immunity^4^. Natural killer cells provide rapid responses to virus-infected and tumor cells^5^. All PBLs work together to protect the body against bacteria, viruses, and other toxins that cause diseases.

Blood-circulating lymphocytes are found in some types of cancers, such as melanoma and colorectal cancer. These lymphocytes migrate into cancer sites and attack tumors^6^. Moreover, the frequency of micronuclei in PBLs is used extensively as a biomarker of chromosomal damage and genome stability in human populations^7^. A high amount of theoretical evidence that supports the causal role of micronucleus induction in cancer development has been accumulated. However, prospective cohort studies are required to validate the usefulness of micronuclei as cancer risk biomarkers. Chimeric antigen receptor (CAR) T-cell therapy, which was awarded a Nobel Prize in 2018, also requires a large amount of PBL cells^8^. Therefore, the process of viably and appropriately isolating PBLs to establish *in vitro* culture systems is crucial for basic research and clinical diagnostics^9^. Isolation of PBLs from whole blood is difficult, and there are serious downsides with existing protocols, e.g., flow sorting, Ficoll-Paque centrifugation, and magnetic particle sorting. Hence, the development of a rapid and safe method to isolate PBLs from whole blood is required^10^.

Venous blood is a ready source of a large number of unstimulated leukocytes, granulocytes, monocytes, and PBLs. By identifying the differences between the relative densities of WBCs circulating in venous blood, lymphocytes can be separated from erythrocytes for use in *ex vivo* studies^11^. Conventionally, a double discontinuous density gradient medium (e.g., Ficoll-Hypaque) is applied to isolate human lymphocytes^12,13^. In this method, collected blood is diluted with phosphate-buffered saline (PBS) and placed on Ficoll-Hypaque. Subsequently, the Ficoll-Hypaque with blood is centrifuged at a low speed for 30 min to isolate the cells according to their cellular density. After centrifugation, the layer in the middle contains lymphocytes.

Immune precipitation is another well-known approach for lymphocyte isolation^14,15^. This method uses antibodies to tag cell surfaces and is a common method for separating lymphocyte subpopulations. The process includes two purification strategies, positive enrichment and negative depletion. Positive enrichment enriches lymphocytes by using an antibody that binds to lymphocytes. Negative depletion depletes nontarget cells and releases lymphocytes. In general, negative depletion is favorable to positive enrichment because the binding of antibodies to target lymphocytes could alter cell features and behaviors during the positive enrichment process. Thus, several commercial kits (e.g., RosetteSep™ Human Total Lymphocyte Enrichment Cocktail) have been designed to enrich lymphocytes from whole blood through negative selection^16–18^. This method aims to remove unwanted venous blood cells by using tetrameric antibody complexes that recognize CD16, CD36, CD66b, and glycophorin A in red blood cells (RBCs). When centrifuged over a buoyant density medium, unwanted cells pellet alongside RBCs. Purified lymphocytes are obtained as a highly enriched population at the interface between the plasma and buoyant density medium.

Another improved lymphocyte isolation method is to directly isolate lymphocytes from human whole blood through immunomagnetic selection. This approach utilizes antibody-immobilized magnetic beads to remove nonlymphocytic cells and enrich lymphocytes. Compared with conventional immune precipitation methods, immunomagnetic selection is faster and easier to use and does not require density gradient centrifugation. Based on the operation principle of immunomagnetic selection, commercial kits (e.g., EasySep™ kit) have been designed to directly isolate highly purified lymphocytes from human whole blood by using immunomagnetic negative selection^19–21^. Magnetic cell sorting not only yields highly pure populations of viable target cells but also requires a relatively short period of time. However, the related reagent is very expensive, and immunomagnetic beads cannot be 100% removed from the final collecting cells.

In 2004, Shashi *et al*. demonstrated lymphocyte isolation by using microfluidic chips^22^. In their experiment, known antibodies were coated on the chamber surfaces of lymphocytes by using silane chemistry or avidin−biotin binding. In addition, poly(ethylene glycol) was coated with the antibodies on the chamber surface to avoid nonspecific binding and improve the reproducibility of lymphocyte cell adhesion. Consequently, this technique proved effective for isolating highly pure lymphocytes from whole blood even when the target cell concentration was low^23^.

In conventional methods, antibodies are essential for lymphocyte sorting. However, antibodies and related chemicals are expensive and thus infeasible for use in basic research^24^. Moreover, conventional processes for blood fractionation are complicated and usually require several steps of pipetting, centrifugation, and labeling, all of which are time consuming, expensive, and require well-trained personnel. Moreover, during manual handling, unwanted artifacts can be introduced into samples, which may affect cell viability. Thus, an automated and operator-independent process is crucial for differentiating blood cells into subpopulations. Microfluidic technology offers opportunities that challenge the performance of macroscale methods^25–29^.

**Figure 1:**
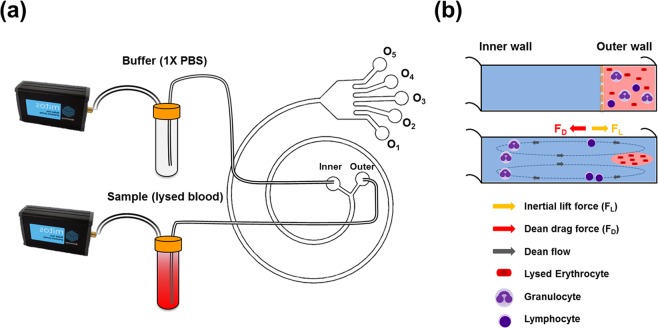
Illustration of the (**a**) setup and (**b**) concept of spiral microfluidic channels for isolating human PBLs rapidly and safely.

In this study, we proposed a simple spiral microfluidic chip for efficient and high-throughput isolation of B and T lymphocytes from a sample with prelysed RBCs (Fig. 1). Compared to most previous spiral inertial microfluidic devices working with syringe pumping systems^30^, two pressure pumps are utilized to drive the sample and sheath buffer, which can easily, rapidly, and steadily operate the optimized sample/sheath ratio to raise separation efficiency in a related nonmeticulous microfluidic device and enable the device to continuously dispose of a large sample. In other parts, this spiral microfluidic platform also takes advantage of properties of other spiral inertial microfluidic devices^31–35^; it does not rely on antibodies or biological markers for labeling cells of interest while isolating lymphocytes but rather enriches lymphocytes through differential physical properties that are intrinsic to lymphocytes and other blood cells.

## Results and Discussion

### Pressure pump for samples

**Figure 2:**
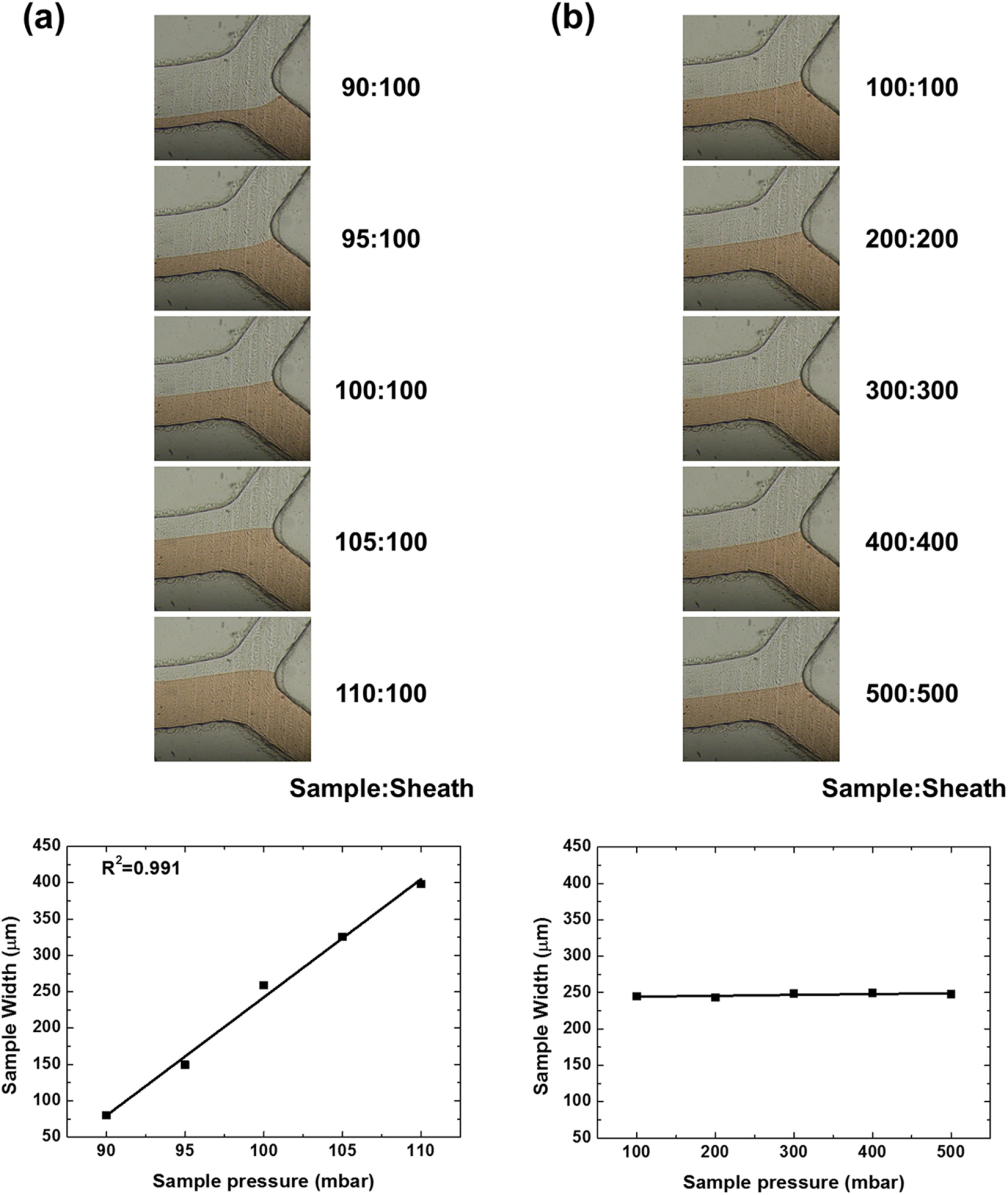
Pressure controls the pigment proportion in the cross section. (**a**) Sheath (transparent 1X PBS) pressure set to a constant value of 100 mbar. The sample (red pigment) pressures from top to bottom are 90, 95, 100, 105, and 110 mbar. (**b**) Sheath pressure increasing in steps of 100 mbar. The pressure ratio of the sample to sheath inlet is 1:1, indicating that the sample width is equal to the sheath width.

The sample was pumped into the spiral microfluidic device at a distinct pressure (while the flow cross-sectional width was maintained at 1:5 = sample:sheath) and collected from the five outlets. A pressure pump can provide steady flow under any pressure condition, and the pressure can be precisely controlled. Compared with other spiral microfluidic devices that narrow down the widths of the sample inlet channel, the two inlets of our device were equal in width and did not block the input. Thus, the input flow in the microfluidic channel remained stable (Fig. 2). In addition, a pressure pumping system can easily, rapidly, and steadily operate an optimized sample/sheath ratio to raise separation efficiency in a related nonmeticulous microfluidic device and can also enable the device to continuously produce a large amount of sample.

### Dean flow in the spiral channel

A Dean flow was generated in the curved microchannel by using a centrifugal vortex, which is also known as a Dean vortex^36^. The distances that the fluid flowed through the inner and outer walls of the curved channel were different because of the unequal velocity in the cross section, and this generated a pressure gradient. The pressure gradient induced a lateral flow field. Thus, a vortex was created in the microchannel, and a recirculating flow was initiated. A lateral rotation phenomenon, known as a Dean vortex, was produced. The formula for the Dean drag force (*F*_*D*_) can be expressed as follows:

$${F}_{D}=3\pi \mu a{U}_{D}$$

$${U}_{D}=1.8\times {10}^{-4}{{D}_{e}}^{1.63}$$

$${D}_{e}=\sqrt{\frac{{D}_{h}}{2{R}_{c}}}\times {R}_{e}$$

where *μ* is the dynamic viscosity, *U*_*D*_ is the local secondary flow velocity field, *D*_*e*_ is the Dean number, *D*_*h*_ is the hydraulic diameter of the microfluidic channel, *R*_*e*_ is the maximum radius of the channel curvature, and *R*_*e*_ is the Reynolds number.

**Figure 3:**
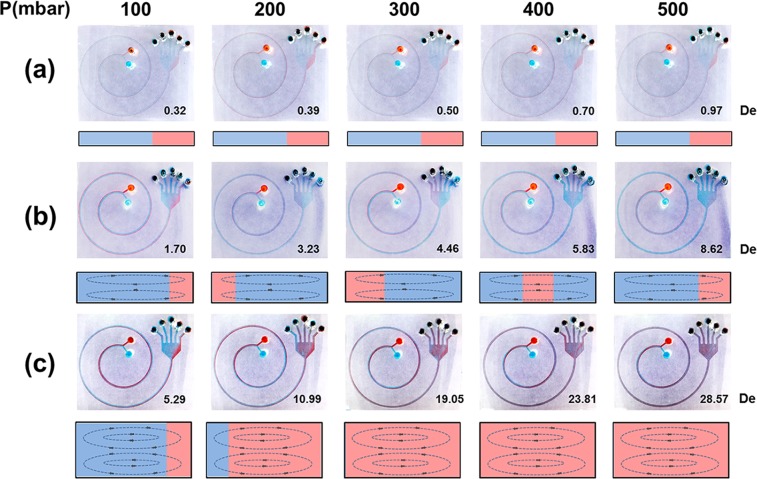
Red and blue pigments were used to simulate flow at different microfluidic channel heights and pumping pressures. (**a**) In the spiral channel with dimensions of 500 (w) × 50 μm^2^ (h), the pressure was 100–500 mbar and the De number was 0.32–0.97. No recirculation cycles were observed under these conditions. (**b**) In the spiral channel with dimensions of 500 (w) × 100 μm^2^ (h), as the pressure gradually increased from 100 to 500 mbar, the De number increased from 1.70 to 8.62. Recirculation cycles were observed and were dependent on pressure. (**c**) In the spiral channel with dimensions of 500  (w) × 200 μm^2^ (h), the De number increased rapidly as the pressure increased gradually (De = 5.29–28.57). Multiple recirculation cycles of the two dyes were obtained.

The presence of Dean flows and their extent in our spiral microchannel were confirmed using two food-dye flows. Dean flows in channels with various aspect ratios and various flow rates were analyzed and are presented in Fig. 3a. In the channels with a 50-µm height, the aspect ratio was high (10), and the Dean number was notably low (*De* = 0.32–0.97) under a pressure range of 100–500 mbar. As presented in Fig. 3a, the dye did not move; the flow was dominated by an inertial lift force, and no significant lateral migration occurred. Therefore, no significant Dean vortices occurred in the channel, and a laminar flow was observed. In each channel with 100-µm height, the aspect ratio was reduced to 5 (Fig. 3b), and the dyes gradually shifted from the outer inlet toward the inner outlet by following the Dean vortices. The magnitudes of these Dean vortices increased with the fluid velocity because of the higher centrifugal force acting on the fluid. The Dean number was 1.70–8.62 under a pressure range of 100–500 mbar. The two dyes switch positions by the time the flow had reached the outlet, thereby indicating a lateral flow. In each channel with a height of 200 µm, the aspect ratio was reduced (2.5; Fig. 3c), and the Dean number was notably high (5.29–28.57) under 100–500 mbar. This caused strong Dean flows and recirculation cycles of the two dyes before the flow reached the outlet. These findings indicated that strong turbulence dominated the lateral flows in the microfluidic channels.

### Inertial lift force (*F*_*L*_) and inertial force ratio (*R*_*f*_) in the spiral channel

Inertial migration is a particle balance phenomenon in microfluidic channels. In inertial migration, the position of a random particle can be manipulated based on the requirements of the experiment being conducted. The net inertial lift force (*F*_*L*_) can be expressed as follows:

$${F}_{L}=\frac{\rho {U}^{2}{a}^{4}}{{{D}_{h}}^{2}}{C}_{L}$$

$${R}_{e}=\frac{\rho U{D}_{h}}{\mu }$$

where *ρ* is the fluidic density, *U* is the maximum velocity, *a* is the particle size, *D*_*h*_ is the hydraulic diameter of the microfluidic channel, *μ* is the dynamic viscosity of the fluid, and *C*_*L*_ is the coefficient of the net inertial lift force, which is related to the Reynolds number (*R*_*e*_) and normalized particle position in the microfluidic channel^37^.

The Dean drag force (*F*_*D*_) and inertial lift force (*F*_*L*_) influence the spiral particle flow in the microfluidic channel. The magnitude of each force contributes to the fluid flow and directly defines the phenomenon that occurs at any spatial position in the microfluidic cross section. To quantify the effects of *F*_*L*_ and *F*_*D*_, the interaction between these two forces was defined in the present study by using the *F*_*L*_*/F*_*D*_ parameter (inertial lift force/Dean force) proposed by Karimi *et al*.^38^, expressed as follows:

$${R}_{f} \sim \frac{{F}_{L}}{{F}_{D}}=\frac{2{R}_{c}{a}^{2}}{{{D}_{h}}^{3}}$$

This parameter describes the force that dominates the flow phenomenon. A spiral microfluidic channel contains three flow regimes: *R*_*f*_ ≫ 1, *R*_*f*_ ≪ 1, and *R*_*f*_ ≈ 1. For *R*_*f*_ ≫ 1, the Dean flow is the secondary flow, and while the lift forces dominate in this regime, the balance of the net lift force and the Dean drag force determine the lateral position of the equilibrium and thus of the particle/cell stream. For *R*_*f*_ ≪ 1, high Dean vortices affect the fluid flow; thus, the particle continues to rotate in the cross section and cannot be focused in the microfluidic channel. For *R*_*f*_ ≈ 1, the Dean drag force and inertial lift force are modified at the equilibrium position of the particle. These factors can help us accurately predict the best conditions to achieve lymphocyte sorting with a balance of lift and Dean drag forces in the device.

**Figure 4:**
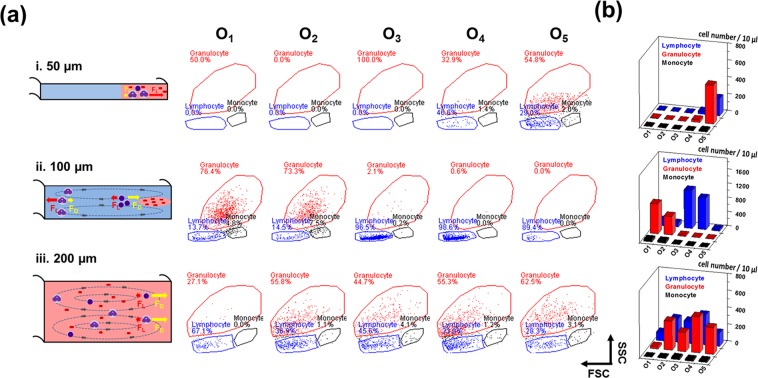
Lymphocyte sorting by using spiral microfluidic chips with various channel heights. Flow cytometry was utilized to confirm the cell components at each outlet. (**a**) Flow cytometry results of the microfluidic channel. The chip heights were (i) 50 µm, (ii) 100 µm, and (iii) 200 µm. The driving sheath pressure was 500 mbar. Lymphocytes are represented by blue lines, granulocytes are represented by dashed red lines, and monocytes are represented by black lines. (**b**) Histogram plot indicating the separation efficiencies of lymphocytes (blue), granulocytes (red), and monocytes (black) by using spiral microfluidic chips of varying channel heights.

Lymphocytes have two flavors: smaller B and T cells, which are 7–8 μm in size, and larger NK cells, which are 12–15 μm in size. The first group overlaps in size with that of RBCs, but this is not an issue if the RBCs are lysed. However, the size of NK cells overlaps with that of basophils (which are granulocytes) and neutrophils (10–12 μm). Therefore, we can only isolate the B and T cells by using our spiral inertial microfluidic device without using surface markers for confirmation. Figure 4 presents the flow cytometry results of lymphocyte sorting via a microfluidic channel with heights (H) of 50, 100, and 200 µm. For H = 50 µm (*R*_*f*_ = 4.5), the inertial lift force dominated the entire system, and no lateral migration was observed. Thus, the cells did not change positions in the spiral microfluidic device, and all the cells remained in the initial sample position (O_5_) (Fig. 4a). When the channel height was increased to 100 µm (*R*_*f*_ = 0.75), the Dean force (*F*_*D*_) and inertial lift force (*F*_*L*_) interacted with each other and were utilized for size-based cell separation. O_1_ and O_2_ primarily contained granulocytes, and lymphocytes were the primary components of the O_3_ (96.5%) and O_4_ (98.6%) outlets (Fig. 4b). When the channel height was 200 µm (*R*_*f*_ = 0.15), the Dean force (*F*_*D*_) acting on the cells was considerably stronger than the inertial force (*F*_*L*_). Thus, the vortex caused by the Dean force dominated the flow status in the microfluidic channel; this implied that the cells could not be focused on a specific position in the microfluidic channel and were broadly separated among the five outlets (Fig. 4c). To determine optimal conditions, lymphocyte sorting was conducted using microfluidic channels with heights (H) of 50, 100, and 200 µm and pumping pressures from 100 to 500 mbar, as presented in the supporting information (Fig. S1). Under optimal conditions (a chip height (H) of 100 µm and a pumping pressure of 500 mbar), high-purity lymphocytes were obtained at O_3_ (99.1%) and O_4_ (98.7%) (Fig. S2).

Optimal separation conditions were observed for a chip with a channel height of 100 µm and pressure within the range of 400 to 500 mbar. The Dean force was sufficiently strong to conduct lateral migration; thus, the particles or cells were arranged at specific positions based on their size. Moreover, because the Dean number was very low (<1 within a pressure range of 100–500 mbar in the device with a channel height of 50 µm [black circle]), the confinement ratio was higher than the critical value of 0.07 (CR = a_p_/h = 0.142 at H = 50 µm) and was supposed to be in focusing mode, but the weak Dean force caused no lateral migration, and the particles or cells could not be brought to positions where *F*_*D*_ and *F*_*L*_ were balanced (Fig. S3). Thus, the particles or cells were not separated under these conditions. Conversely, for the channel with a height of 200 µm, the Dean number was considerably higher than the corresponding values in the channels with other heights. In this case, the Dean force was very large and could not be balanced by the internal force. The lateral flow caused the particles or cells to rotate in the microfluidic cross-section; thus, the particles or cells could not be separated.

The results of a previous experiment (Fig. 3) revealed that optimal separation was observed using a channel with a height of 100 µm and a pressure of 400 or 500 mbar (middle Dean vortex). Based on the results presented in Fig. S4, the Dean number was very low (<1) at any pressure in the device at a channel height of 50 µm (black circle). Although the confinement ratio was higher than the critical value of 0.07 (CR = a_p_/h = 0.142 at H = 50 µm), the low Dean number indicates that no lateral migration occurred in the device. This implies that the particles or cells did not separate. The Dean number increased at a channel height of 100 µm. The confinement ratio was 0.072 (>0.07) under this condition, and the Dean number was sufficiently high to cause lateral migration under high pressure. Thus, the particles or cells had an excellent opportunity to separate to specific positions based on their sizes. The Dean number rapidly increased at a channel height of 200 µm and was sufficiently high to cause a lateral flow to drive the particles or cells to migrate in the microfluidic cross-section. Although most of the results indicated that a spiral device with a channel height of 200 µm could accomplish size-based cell separation, some evidence suggests that this method is ineffective. The confinement ratio for the channel height of 200 µm was approximately 0.042 (<0.07); thus, particles were unable to separate appropriately.

### Safe lymphocyte sorting

**Figure 5:**
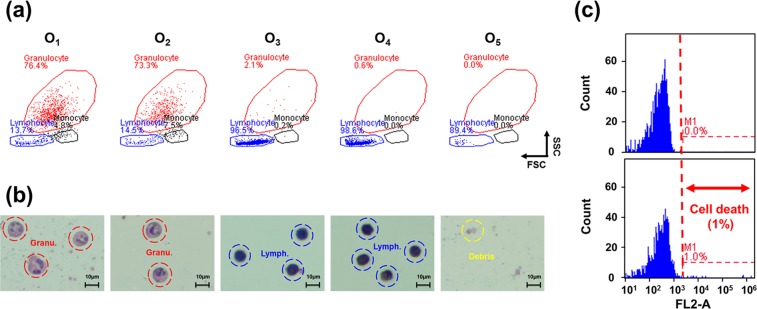
(**a**) Flow cytometry results revealed that lymphocytes were obtained at O_3_ and O_4_ (~90%) and granulocytes were obtained at O_1_ and O_2_ (~70%). (**b**) OM images of cells stained using Liu’s staining approach that were obtained at each outlet. Granulocytes are represented by dotted red circles, lymphocytes are represented by dotted blue circles, and debris is represented by dotted yellow circles. (**c**) Regarding the percentage of living lymphocytes, approximately 1% of the lymphocytes were dead.

To understand whether the MFC purification system damaged lymphocytes, purified lymphocytes collected from O_4_ (Fig. 5a) were selected for a cell viability test. OM cell images were utilized to confirm the types of cells collected (Fig. 5b). As presented in Fig. 5b, the cells isolated from O_3_ and O_4_ were typically lymphocytes. Moreover, propidium iodide (PI) staining^39^, a popular red-fluorescent nuclear and chromosome counterstain, was utilized to confirm the cell conditions. Notably, PI cannot be used for living cells and is widely utilized to stain dead cells. Flow cytometry was utilized to count the percentage of dead lymphocytes. As presented in Fig. 5c, only 1% of the lymphocytes were dead, leaving 99% alive; this result clearly reveals that a spiral microfluidic channel is safe for rapid cell sorting. While the viability of cells following separation in a spiral device is provided here, whether flow may trigger some function of lymphocytes or other abnormal activation may need further detailed research.

## Conclusion

Rapid and safe isolation of lymphocytes from whole blood is a fundamentally essential task. In this study, we successfully developed a microfluidic spiral chip for efficient and high-throughput isolation of lymphocytes from a sample with prelysed RBCs. Like other hydrodynamics-based devices^44–48^, this spiral microfluidic platform that isolates lymphocytes does not rely on antibodies or biological markers for labeling cells of interest but rather enriches cells by using differential physical properties that are intrinsic to lymphocytes and other blood cells. By conducting tests with spiral microfluidic channels with various channel heights, we determined that an appropriate Dean vortex is crucial for lymphocyte cell isolation. Based on the results obtained from individual experiments, spiral channels with heights of 100 and 500 µm and a sheath buffer pressure under 500 mbar were employed to separate lymphocytes. The device was also used to achieve high-throughput (~1.3 × 10^5^ cells/min) separation of lymphocytes with high viability (>99%). The comparison numbers presented in Table 1 reveal that our device provided rapid, label-free, high-throughput, and safe lymphocyte separation. This design could easily be integrated into existing LOC systems that require high-throughput B and T lymphocyte separation and might integrate with other sensing platforms^49^ to achieve automation and diagnosis^50–52^.

**Table 1:** Comparison of lymphocytes isolated using various methods.

Method	Flow rate (μL/min)	Throughput (cells/min)	Purity (%)	Total time (min)	Cost	Reference
**Traditional**						
Surface modification	~10	~1.6*10^5^	~95	~90	High	^22^
Cell adhesion	~50	~2.5*10^5^	~60	~40	Low	^40^
Density gradient	~167	~2*10^5^	~90	~70	High	^41^
**Microfluidic**						
Immunomagnetic	~83.3	~2.9*10^3^	~95	~170	High	^42^
Hydrodynamic filtration	~10	~6*10^3^	~90	~15	High	^43^
Deterministic Lateral Displacement	~0.2	~6*10^4^	~90	~40	Low	^44^
Step pattern filtration	~0.1	~7.7*10^3^	~90	~60	Low	^45^
Dean drag force & Inertial lift force	~1700	~1.3*10^5^	97 ± 2	~15	Low	our device

## Materials and Methods

### Device design and characterization

The proposed device comprises two inlets (an outer wall and inner wall) and five outlets (O_1_ to O_5_). The total length of the channel is approximately 9 cm, and the surface area is approximately 4 cm^2^. The small-sized microdevice can be used for high-throughput cell sorting in clinical diagnostic pretreatment. The cross-sectional design of the microchannel is crucial for size-based cell separation and offers a convenient method for exploring the interrelationships between Dean number (*De*), aspect ratio (*AR*), and confinement ratio (*CR*). In this study, we used three different geometries, set the channel width as constant (W = 500 µm), and employed channel height as a variable (H = 50, 100, 200 µm). Changes due to these three designs were analyzed to obtain an optimal condition for size-based cell separation.

The device utilized in this study contains a spiral geometry with two inlets 500 μm in diameter, five outlets 500 μm in diameter separated by 500-μm-long spacing, and various channels with heights of 50–200 µm. The maximum radius of curvature of the spiral was 1 cm, and the total length of each microchannel was approximately 9 cm. The spiral microfluidic chip was fabricated using PDMS, and the fabrication process is illustrated in Fig. S5. The molds of the spiral microchannels were prepared on a PMMA substrate through CNC machine milling rather than photolithography because CNC milling enabled us to easily prepare molds of varying channel height. The PMMA substrate was milled using the CNC machine to obtain the spiral microfluidic pattern shown in Fig. S5. PDMS was poured into the microfluidic mold and baked for 50 min at 80 °C for PDMS curing. Subsequently, the PDMS was peeled off the mold, and inlet and outlet holes were punched. Finally, PDMS was bound to glass slides through oxygen plasma pretreatment (the detailed fabrication procedure is presented in the materials and methods section)^53^.

### Fabrication

The spiral microfluidic channel was fabricated using polydimethylsiloxane polymer (PDMS) (Sylgard 184 Elastomer Kit, Dow Corning Corporation, USA) and poly(methyl methacrylate) (PMMA) molds as templates. The PMMA molds were fabricated by micromilling (EGX-400 engraving machine, Roland, USA) to form a special cross-sectional design. To fabricate the molds, a mixture with a 10:1 ratio of silicone elastomer (A agent) to curing agent (B agent) was used. This mixture was baked at 80 °C for 1 h in a precision drying oven (DOS 300, Dengyng, Taiwan). Subsequently, the PDMS was peeled off the PMMA mold, and inlet and outlet holes were created using a biopsy punch 1.5 mm in diameter (Ted Pella Inc., USA). Finally, the PDMS slab was irreversibly bonded to a standard glass slide by infiltrating the device in oxygen plasma (Zepto plasma, Diener, DE) under 5 N oxygen pressure of 1 mbar (0.5 L/h^−1^) at 60 W for 60 s.

### Experimental setup

The spiral microfluidic channel was mounted on the stage of an inverted microscope (IVM-2A, SAGE Vision, TW) equipped with a complementary metal-oxide-semiconductor camera (EOS 700D, Canon, JP). A retrofit 50-mL tube (Corning® CentriStar™, Sigma-Aldrich, USA) was used as a pressure vessel to connect the pressure pump (Mitos Fluika low pressure pump, Dolomite, UK) and microfluidic channel with 1- and 5-mm (outer diameter [OD]) Teflon tubes (Fluo-Tech Corp., TW). Cell mixtures were pumped into the spiral microfluidic device, and separated cells were collected with 1.5-mL tubes. Subsequently, flow cytometry analysis was applied to the sample to investigate the pressure that constituted the optimal condition for size-based cell separation. Moreover, a digital microscope (DVM6, Leica, DE) was utilized to observe cell morphology and approximately estimate the ratios of various WBC types in each outlet sample.

### In-chip color experiments

The operational principle of the embedded spinneret was visualized using two colored fluids, as displayed in an optical microscopy image (Canon 650D). Blue (E133) and red (E100) food coloring (Sigma-Aldrich, Inc., US) were separately dispersed in water by ultrasonication (Sage Vision Co., Ltd, TW) at a water-to-dye ratio of 1:2 (w/w). A system comprising two pressurized vessels was connected to the inlets of the microfluidic chip via glued tubing. The pressure was adjusted manually until a stable flow profile was obtained. The spiral microfluidic channel was mounted on the stage of an inverted microscope (dimensions: 25 × 75 mm^2^; Plain, Fea, TW) equipped with a digital camera (Sony, DSC-RX 10 II, JP)

### Test sample preparation

Human whole blood samples were obtained from healthy donors under the approval of the ethics committee according to a protocol permitted by the Institutional Review Board (IRB) (NCTU-REC-107-071). In addition, informed consent forms were provided to all participants in our research. Fresh human blood samples were collected from three healthy donors by using a vacutainer tube that contained ethylenediaminetetraacetic acid (EDTA), an anticoagulant agent. EDTA was used to bind calcium ions in the blood sample; this mechanism can prevent aggregation of RBCs. To separate lymphocytes and RBCs based on their size (normally, lymphocytes and RBCs are approximately equal in size), the whole blood sample was lysed using a 1X RBC lysis buffer (with 0.15 M NH_4_Cl, 1 mM KHCO_3_, and 0.1 mM K_2_EDTA) (ratio: 1:10) for 7 min. Then, the reaction was quenched using PBS (1X PBS). Subsequently, a high-speed refrigeration centrifuge (320 R, HETTICH, DE) was used to remove most of the lysed RBCs in the supernatant; the leukocytes were washed using 1X PBS. Finally, the sample was pumped into the spiral microfluidic device to separate the different types of WBCs.

### Flow cytometry

After conducting size-based cell separation by using a spiral microfluidic channel, all the outlet samples were collected, and the separation efficiency was confirmed using flow cytometry (Accuri™ C6 Plus System, BD Biosciences, USA) to detect the ratios of all types of WBCs. The characteristics of WBC size and granularity were then used to distinguish between WBC types. In flow cytometry, after the particles or cells had been excited using a laser, a 0.5°–5° forward scatter (FSC) and 15°–150° side scatter (SSC) were generated. The FSC was proportional to the size of the cells, whereas the SSC was directly proportional to the granularity of the particles or cells. After verification of the lymphocyte ratios, propidium iodide (PI) was utilized to dye the cell nuclei.

The authors confirm that human whole blood samples were obtained from healthy donors under the approval of the Research Ethics Committee for Human Subject Protection, National Chiao Tung University, according to a protocol permitted by the Institutional Review Board (IRB) (NCTU-REC-107-071).

## Supplementary information

Supporting information

## References

[1] Hayday A, Theodoridis E, Ramsburg E, Shires J (2001). Intraepithelial lymphocytes: exploring the Third Way in immunology. Nat Immunol.

[2] Gros A (2016). Prospective identification of neoantigen-specific lymphocytes in the peripheral blood of melanoma patients. Nat Med.

[3] LeBien TW, Tedder TF (2008). B lymphocytes: how they develop and function. Blood.

[4] Wood KJ, Sakaguchi S (2003). Regulatory T cells in transplantation tolerance. Nature Reviews Immunology.

[5] Vivier E, Tomasello E, Baratin M, Walzer T, Ugolini S (2008). Functions of natural killer cells. Nat Immunol.

[6] Bellone M, Calcinotto A (2013). Ways to enhance lymphocyte trafficking into tumors and fitness of tumor infiltrating lymphocytes. Frontiers in oncology.

[7] Biswas SK, Mantovani A (2010). Macrophage plasticity and interaction with lymphocyte subsets: cancer as a paradigm. Nat Immunol.

[8] Davila M. L., Riviere I., Wang X., Bartido S., Park J., Curran K., Chung S. S., Stefanski J., Borquez-Ojeda O., Olszewska M., Qu J., Wasielewska T., He Q., Fink M., Shinglot H., Youssif M., Satter M., Wang Y., Hosey J., Quintanilla H., Halton E., Bernal Y., Bouhassira D. C. G., Arcila M. E., Gonen M., Roboz G. J., Maslak P., Douer D., Frattini M. G., Giralt S., Sadelain M., Brentjens R. (2014). Efficacy and Toxicity Management of 19-28z CAR T Cell Therapy in B Cell Acute Lymphoblastic Leukemia. Science Translational Medicine.

[9] Morsy MA (2005). Isolation, purification and flow cytometric analysis of human intrahepatic lymphocytes using an improved technique. Lab Invest.

[10] Lin YL (2016). *In vivo* amelioration of endogenous antitumor autoantibodies via low-dose P4N through the LTA4H/activin A/BAFF pathway. P Natl Acad Sci USA.

[11] Klein IA (2011). Translocation-Capture Sequencing Reveals the Extent and Nature of Chromosomal Rearrangements in B Lymphocytes. Cell.

[12] Zhang Y, Huang SD, Gong DJ, Qin YH, Shen QA (2010). Programmed death-1 upregulation is correlated with dysfunction of tumor-infiltrating CD8(+) T lymphocytes in human non-small cell lung cancer. Cell Mol Immunol.

[13] Molldrem JJ (2000). Evidence that specific T lymphocytes may participate in the elimination of chronic myelogenous leukemia. Nat Med.

[14] Boczkowski D, Nair SK, Nam JH, Lyerly HK, Gilboa E (2000). Induction of tumor immunity and cytotoxic T lymphocyte responses using dendritic cells transfected with messenger RNA amplified from tumor cells. Cancer Res.

[15] Rodriguez PC, Quiceno DG, Ochoa AC (2007). L-arginine availability regulates T-lymphocyte cell-cycle progression. Blood.

[16] Busch R (2004). Isolation of peripheral blood CD4+ T cells using RosetteSep™ and MACS™ for studies of DNA turnover by deuterium labeling. Journal of Immunological Methods.

[17] Hotchkiss RS (2006). TAT-BH4 and TAT-Bcl-xL Peptides Protect against Sepsis-Induced Lymphocyte Apoptosis *In Vivo*. The Journal of Immunology.

[18] Dosiou C (2008). Expression of membrane progesterone receptors on human T lymphocytes and Jurkat cells and activation of G-proteins by progesterone. J Endocrinol.

[19] Spaggiari GM (2008). Mesenchymal stem cells inhibit natural killer-cell proliferation, cytotoxicity, and cytokine production: role of indoleamine 2,3-dioxygenase and prostaglandin E2. Blood.

[20] Thompson EA, Beura LK, Nelson CE, Anderson KG, Vezys V (2016). Shortened Intervals during Heterologous Boosting Preserve Memory CD8 T Cell Function but Compromise Longevity. J Immunol.

[21] Mian MF, Lauzon NM, Andrews DW, Lichty BD, Ashkar AA (2010). FimH Can Directly Activate Human and Murine Natural Killer Cells via TLR4. Mol Ther.

[22] Murthy SK, Sin A, Tompkins RG, Toner M (2004). Effect of flow and surface conditions on human lymphocyte isolation using microfluidic chambers. Langmuir.

[23] Lenshof A, Laurell T (2010). Continuous separation of cells and particles in microfluidic systems. Chem Soc Rev.

[24] Sarkar, A., Hou, H. W., Mahan, A. E., Han, J. & Alter, G. Multiplexed Affinity-Based Separation of Proteins and Cells Using Inertial Microfluidics. *Sci Rep-Uk* **6**, 10.1038/Srep23589 (2016). 10.1038/srep23589 PMC4812309 27026280

[25] Yamada M, Seki M (2005). Hydrodynamic filtration for on-chip particle concentration and classification utilizing microfluidics. Lab Chip.

[26] Li BR (2013). An Ultrasensitive Nanowire-Transistor Biosensor for Detecting Dopamine Release from Living PC12 Cells under Hypoxic Stimulation. J Am Chem Soc.

[27] Lin TY (2013). Improved silicon nanowire field-effect transistors for fast protein-protein interaction screening. Lab Chip.

[28] Kitson PJ, Rosnes MH, Sans V, Dragone V, Cronin L (2012). Configurable 3D-Printed millifluidic and microfluidic ‘lab on a chip’ reactionware devices. Lab Chip.

[29] Yang Chiao-Hsun, Hsieh Yu-Ling, Tsou Ping-Hsien, Li Bor-Ran (2019). Thermopneumatic suction integrated microfluidic blood analysis system. PLOS ONE.

[30] Guan, G. F. *et al*. Spiral microchannel with rectangular and trapezoidal cross-sections for size based particle separation. *Sci Rep-Uk* **3**, 10.1038/Srep01475 (2013). 10.1038/srep01475 PMC3600595 23502529

[31] Bhagat AAS, Kuntaegowdanahalli SS, Papautsky I (2008). Continuous particle separation in spiral microchannels using dean flows and differential migration. Lab Chip.

[32] Kuntaegowdanahalli SS, Bhagat AAS, Kumar G, Papautsky I (2009). Inertial microfluidics for continuous particle separation in spiral microchannels. Lab Chip.

[33] Martel Joseph M., Toner Mehmet (2012). Inertial focusing dynamics in spiral microchannels. Physics of Fluids.

[34] Choi K (2018). Negative Selection by Spiral Inertial Microfluidics Improves Viral Recovery and Sequencing from Blood. Anal Chem.

[35] Kwon, T. *et al*. Microfluidic Cell Retention Device for Perfusion of Mammalian Suspension Culture. *Sci Rep-Uk* **7**, 10.1038/S41598-017-06949-8 (2017). 10.1038/s41598-017-06949-8 PMC5532224 28751635

[36] Di Carlo D (2009). Inertial microfluidics. Lab Chip.

[37] Bhagat AAS, Hou HW, Li LD, Lim CT, Han JY (2011). Pinched flow coupled shear-modulated inertial microfluidics for high-throughput rare blood cell separation. Lab Chip.

[38] Karimi A., Yazdi S., Ardekani A. M. (2013). Hydrodynamic mechanisms of cell and particle trapping in microfluidics. Biomicrofluidics.

[39] Riccardi C, Nicoletti I (2006). Analysis of apoptosis by propidium iodide staining and flow cytometry. Nature protocols.

[40] Lamvik JO (1966). Separation of Lymphocytes from Human Blood. Acta Haematologica.

[41] Maurer HR (1979). Colony Growth of Human and Mouse Granulocytes, Macrophages, Lymphocyte-T and Lymphocyte-B in Agar Capillaries and Its Application to the Isolation of Growth Stimulating and Inhibiting Factors. Exp Hematol.

[42] Davis JA (2006). Deterministic hydrodynamics: Taking blood apart. P Natl Acad Sci USA.

[43] Darabi Jeff, Guo Chuan (2013). On-chip magnetophoretic isolation of CD4 + T cells from blood. Biomicrofluidics.

[44] Choi S, Song S, Choi C, Park JK (2007). Continuous blood cell separation by hydrophoretic filtration. Lab Chip.

[45] Mizuno M (2013). Magnetophoresis-Integrated Hydrodynamic Filtration System for Size- and Surface Marker-Based Two-Dimensional Cell Sorting. Anal Chem.

[46] Yamada M, Seko W, Yanai T, Ninomiya K, Seki M (2017). Slanted, asymmetric microfluidic lattices as size-selective sieves for continuous particle/cell sorting. Lab Chip.

[47] Hou, H. W. *et al*. Rapid and label-free microfluidic neutrophil purification and phenotyping in diabetes mellitus. *Sci Rep-Uk* **6**, 10.1038/Srep29410 (2016). 10.1038/srep29410 PMC4933935 27381673

[48] Bhagat AA, Hou HW, Li LD, Lim CT, Han J (2011). Pinched flow coupled shear-modulated inertial microfluidics for high-throughput rare blood cell separation. Lab Chip.

[49] Li BR, Shen MY, Yu HH, Li YK (2014). Rapid construction of an effective antifouling layer on a Au surface via electrodeposition. Chem Commun.

[50] Chang CY, Li BR, Li YK (2016). An L-ascorbate-6-phosphate lactonase from Streptococcus pneumoniae ATCC 49136 strain reveals metallo-beta-lactamase activity. Int J Antimicrob Ag.

[51] Lin Pei-Heng, Huang Sheng-Cih, Chen Kuang-Po, Li Bor-Ran, Li Yaw-Kuen (2018). Effective Construction of a High-Capacity Boronic Acid Layer on a Quartz Crystal Microbalance Chip for High-Density Antibody Immobilization. Sensors.

[52] Chang Chia-Yu, Lin Hui-Jen, Li Bor-Ran, Li Yaw-Kuen (2016). A Novel Metallo-β-Lactamase Involved in the Ampicillin Resistance of Streptococcus pneumoniae ATCC 49136 Strain. PLOS ONE.

[53] Li BR (2013). Biomolecular recognition with a sensitivity-enhanced nanowire transistor biosensor. Biosens Bioelectron.

