# Supplementary material for: Rapid and Safe Isolation of Human Peripheral Blood B and T Lymphocytes through Spiral Microfluidic Channels
Source: Sci Rep. 2019 May 31;9:8145. doi: 10.1038/s41598-019-44677-3 (PMC6544655; doi:10.1038/s41598-019-44677-3)
Supplement: Supplementary file 1 — Supporting information [file 41598_2019_44677_MOESM1_ESM.pdf]

Supporting information

Rapid and Safe Isolation of Human Peripheral Blood B and T Lymphocytes through Spiral Microfluidic Channels

Po-Lin Chiu,[1] Chun-Hao Chang,[1] Yu-Ling Lin,[2,3] Ping-Hsien Tsou,[3,4] and Bor-Ran Li [1, 5]*

1. Institute of Biomedical Engineering, College of Electrical and Computer Engineering, National Chiao Tung University, Hsinchu, Taiwan.
2. Agricultural Biotechnology Research Center, Academia Sinica, Taipei, Taiwan
3. Department of Biological Science and Technology, College of Biological Science and Technology, National Chiao Tung University, Hsinchu, Taiwan.
4. Division of Chest Medicine, Department of Internal Medicine, National Taiwan University Hospital, Hsinchu, Taiwan.
5. Center for Emergent Functional Matter Science, National Chiao Tung University, Hsinchu, Taiwan, ROC

* To whom correspondence should be addressed.

Dr. Bor-Ran Li
Address: Institute of Biomedical Engineering, National Chiao Tung University,
1001 Ta-Hseh Rd. Hsinchu, Taiwan
Tel No: 886-3-5712121 ext. 54051
Fax No: 886-3-5165993
E-mail: liborran@g2.nctu.edu.tw

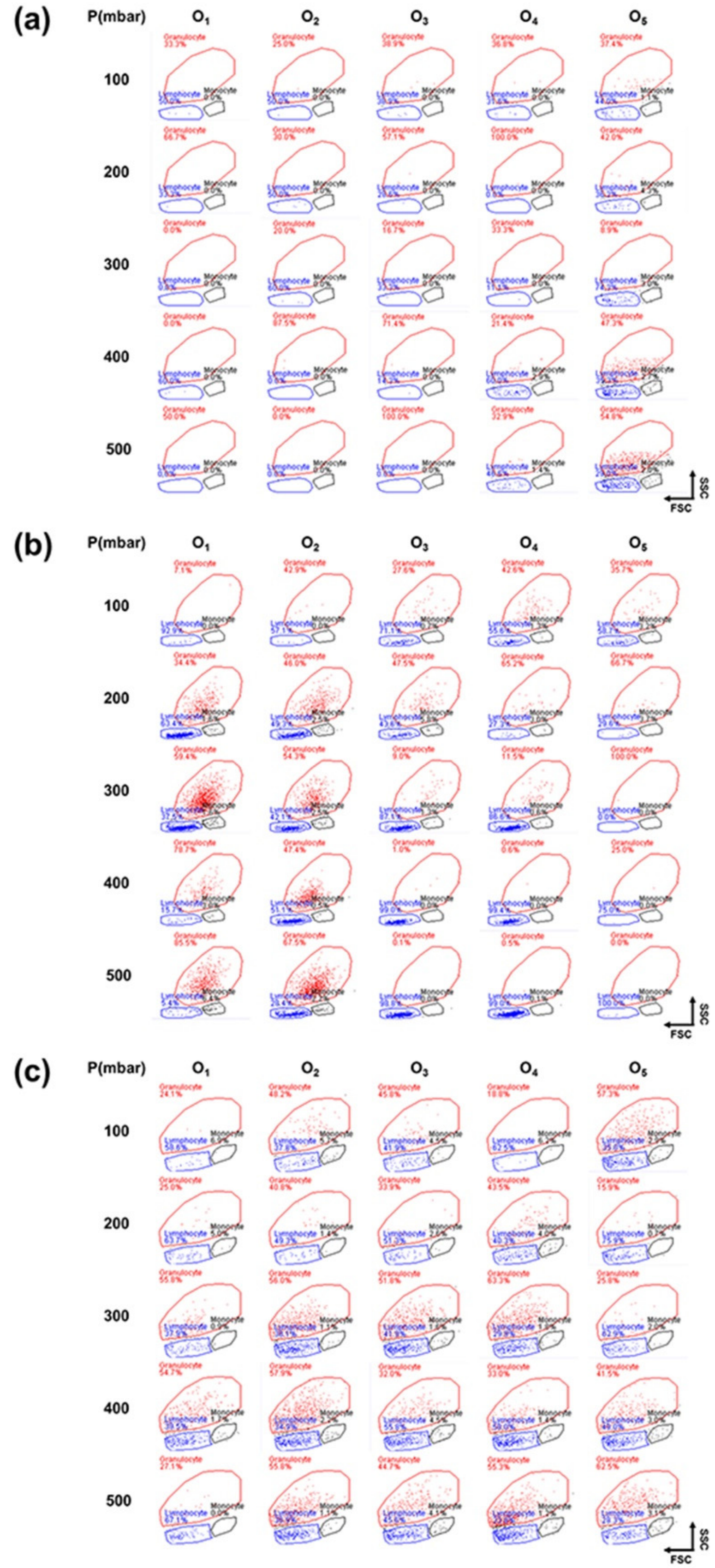

Fig. S1 *Lymphocyte sorting by spiral microfluidic chips with channel heights of (a) 50 μm, (b) 100 μm, and (c) 200 μm.*

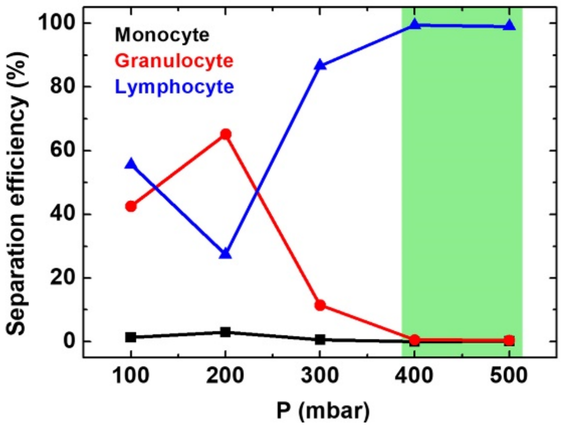

Fig. S2 *Flow rate characterization as a function of neutrophil sorting in outlet 3 of the spiral device. Optimal separation was achieved at a chip height (H) of 100 μm and a pumping pressure over 500 mbar.*

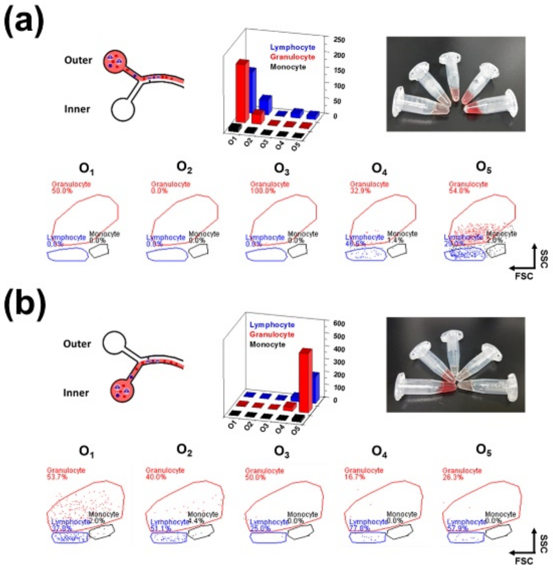

Fig. S3 *Exchange of blood and buffer inlet in the chip. (a) Blood cells are injected via the outer inlet, and the sheath buffer is injected via the inner inlet. The cells do not separate and are mainly collected at* O_4 *and* O_5*. (b) The sheath buffer is injected via the outer inlet, and the blood cells are injected via the inner inlet. The cells do not separate and are mainly collected at* O_1 *and* O_2*. The results indicate that inertial force (*F_L*) dominates the movement of the cells and that no lateral migration occurs.*

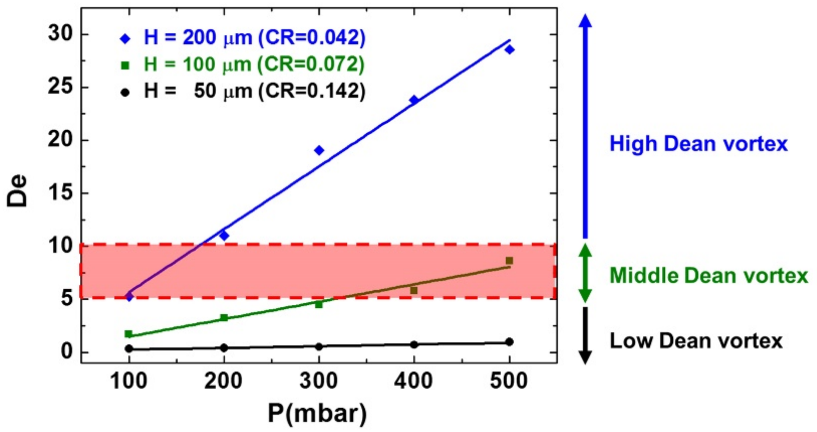

Fig. S4 *Dean number (De) characterization as a function of lymphocyte sorting at various channel heights and pressure values in the spiral microfluidic channel.*

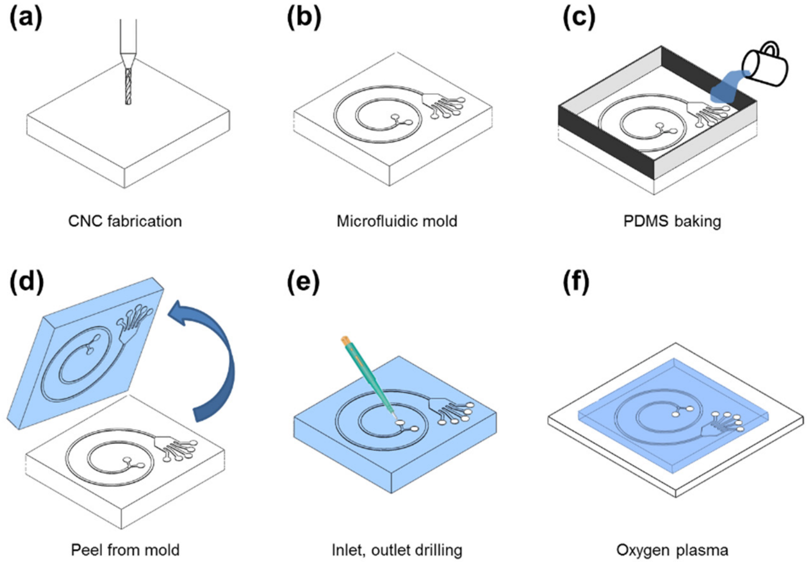

Fig. S5 *Fabrication process steps for PDMS molding. (a), (b) Mold preparation via CNC milling. (c) Pouring and baking of PDMS at 80 °C for 50 min. (d) Peeling off the cured PDMS. (e) Punching inlet and outlet holes. (f) Binding PDMS to glass slides through oxygen plasma pretreatment.*
